# Preparation and Synergistic Activation Mechanism of Cemented Backfill Materials Utilizing MSWI Fly Ash and Low-Titanium Slag

**DOI:** 10.3390/ma19081551

**Published:** 2026-04-13

**Authors:** Bo Su, Jie Chi, Siqi Zhang, Jia Li, Keqing Li, Xingyang Xu, Wen Ni

**Affiliations:** 1 Institute of Minerals Resources, University of Science and Technology Beijing, Beijing 100083, China; ustb_subo@163.com (B.S.);; 2 School of Civil and Resource Engineering, University of Science and Technology Beijing, Beijing 100083, China; 3 China-Zambia Belt and Road Joint Laboratory on Green and Safe Development of Mineral Resources, School of Resources and Safety Engineering, University of Science and Technology Beijing, Beijing 100083, China

**Keywords:** low-titanium slag, MSWI fly ash, composite activation, low-carbon cementitious materials, cemented backfill materials

## Abstract

A low-titanium-slag-based multi-solid-waste cementitious system was developed for cemented paste backfill. The cementitious binder was prepared from low-titanium slag (LTS), steel slag (SS), municipal solid waste incineration (MSWI) fly ash, and flue gas desulfurization gypsum (FGDG), while lead–zinc tailings were used as the aggregate for backfill materials preparation. The activation of low-titanium slag, proportion optimization, and strength development mechanisms were systematically investigated. Mechanical grinding effectively activated low-titanium slag, and its activity index reached 108% after 90 min of grinding at 28 d. Steel slag alone could not fully activate low-titanium slag in the ternary system, whereas the incorporation of MSWI fly ash significantly enhanced the synergistic activation effect. The quaternary system with 40% MSWI fly ash replacement showed higher cumulative heat release and better later-age strength. The optimum backfill proportion was a solid mass concentration of 81% with a binder-to-tailings ratio of 1:4, yielding a 28 d compressive strength of 11.07 MPa with satisfactory flowability and setting behavior. Microstructural results indicated that the continuous formation of ettringite and gel phases promoted pore refinement and matrix densification. Moreover, the leaching concentrations of Pb, Zn, Cr, and soluble Cl were all below the relevant groundwater quality limits. These results demonstrate a feasible route for the high-value co-utilization of low-titanium slag and MSWI fly ash in cemented backfill materials.

## 1. Introduction

With the ongoing implementation of carbon neutrality strategies and the development of green mining, cemented paste backfill has become an important approach for sustainable underground mining because of its advantages in large-scale tailings utilization, stope support, and mitigation of surface environmental disturbance [1]. However, conventional cemented paste backfill materials remain highly dependent on ordinary Portland cement, which results in high economic cost, substantial carbon emissions, and considerable resource consumption [2]. In this context, the development of low-carbon backfill binders derived from industrial solid wastes has attracted increasing attention, and the substitution of conventional cementitious materials with metallurgical slags, chemical by-products, and other industrial residues has been widely recognized as an effective strategy for reducing environmental burdens and improving solid waste valorization [3]. Moreover, the performance of cemented paste backfill is governed not only by compressive strength, but also by the coupled effects of flowability, setting behavior, cost, and long-term serviceability. Accordingly, mixture design for backfill materials is essentially a multi-objective optimization problem [4,5]. Previous studies have further shown that the fresh properties and long-term evolution of backfill materials strongly affect pipeline transportability, placement stability, and in-service reliability [6,7]. Therefore, developing cemented paste backfill binders dominated by low-reactivity industrial solid wastes, while ensuring low carbon intensity and adequate engineering applicability, has become a major research priority [8,9].

Low-titanium slag is a bulk industrial residue generated during titanium smelting and contains considerable amounts of Ca, Si, and Al, indicating its potential as a cementitious precursor [10]. Nevertheless, unlike highly reactive ground granulated blast furnace slag, low-titanium slag and related titanium-bearing slags usually exhibit high crystallinity and stable mineral structures, which makes their latent hydraulic reactivity difficult to activate under conventional conditions [11]. Existing studies have shown that titanium slag is not completely inert in low-carbon cement-based systems; rather, its contribution to hydration depends strongly on particle refinement, activation conditions, and the characteristics of the blended matrix [12,13]. Investigations on slurry cutoff wall materials, grouting materials, and high-temperature cementitious systems have further demonstrated that titanium-bearing slags can participate in hydration or modify structural development once the reaction environment is properly tailored [14,15]. However, recent evidence from vanadium–titanium slag/blast furnace slag blended systems also suggests that titanium-bearing slags may suppress heat release and strength development when activation is insufficient [16]. These findings indicate that the key challenge in the high-value utilization of low-titanium slag lies not in its elemental composition, but in the establishment of an effective activation pathway that can transform it from a low-reactivity residue into a viable cementitious precursor.

Synergistic activation using multiple solid wastes offers a promising route for enhancing the utilization efficiency of low-reactivity precursors. Steel slag can provide alkalinity and Ca sources, whereas flue gas desulfurization gypsum can continuously supply sulfate ions. When combined with blast furnace slag, these components can establish a reaction environment favorable for the formation of AFt and C-(A)-S-H gel, thereby improving both mechanical performance and volumetric stability [17]. In high-steel-slag cemented backfill systems, the synergistic interactions among steel slag, blast furnace slag, and desulfurization gypsum have been shown to play a decisive role in controlling reaction pathways and engineering performance [18]. From this perspective, introducing low-titanium slag into a steel slag–desulfurization gypsum system, while further incorporating additional solid wastes to provide reactive Al species and soluble salts, may offer an effective means of overcoming the insufficient activation associated with the standalone use of low-titanium slag.

MSWI fly ash is another typical solid waste that simultaneously poses environmental risks and exhibits resource utilization potential. On the one hand, MSWI fly ash is enriched in soluble chlorides, heavy metals, and toxic trace constituents, which may cause substantial environmental hazards if improperly managed [19]. On the other hand, its relatively high alkalinity, soluble salt content, and reactive Al species also give it the potential to participate in cementitious reaction regulation [20]. Recent studies and reviews have increasingly suggested that MSWI fly ash should not be regarded solely as a hazardous residue requiring stabilization; rather, when co-processed with Al- and Si-bearing solid wastes, it can contribute to pollutant immobilization and material valorization through phase reconstruction, ionic supply, and mineral encapsulation [21,22]. Further investigations on alkali-activated systems have shown that the strength development of MSWI fly ash-based matrices, as well as the immobilization of heavy metals and chloride ions, is highly dependent on activation conditions, gel structure, and the formation of Al-bearing phases such as ettringite and Friedel’s salt [23]. Application studies on ecological concrete and pavement bricks have also demonstrated the practical feasibility of incorporating MSWI fly ash into construction materials at suitable dosages, provided that its environmental risks are effectively controlled through rational system design [24,25]. Accordingly, treating MSWI fly ash as a synergistic activation component rather than merely a pollution-bearing waste may provide a new opportunity for simultaneously promoting low-titanium slag reactivity and immobilizing hazardous constituents [26].

Despite these advances, several key issues remain unresolved. First, the role of low-titanium slag in multi-solid-waste cementitious systems has not yet been clearly established, particularly when it serves as the principal precursor, and its reactivity-release behavior and contribution to strength development remain insufficiently understood. Second, in low-titanium-slag-based systems, the function of MSWI fly ash has not been clearly clarified, namely whether it primarily acts as an environmental burden, a waste-derived activator, or both. Third, for underground cemented paste backfill applications, mixture design should not be evaluated solely in terms of binder strength, but should simultaneously account for multiple constraints, including flowability, setting time, strength grade, and leaching safety, so as to establish a direct linkage between reaction behavior and engineering applicability.

In view of the above, this study developed a low-titanium-slag-based multi-solid-waste cementitious system, in which low-titanium slag, steel slag, MSWI fly ash, and FGDG were used to formulate the binder, while lead–zinc tailings were used as the aggregate for cemented paste backfill material preparation. The work systematically investigated the activation of the latent cementitious reactivity of low-titanium slag, the proportion optimization of the corresponding cemented paste backfill, and the associated strength development mechanisms. First, the reactivity enhancement of low-titanium slag induced by mechanical grinding was evaluated. Subsequently, a low-titanium slag–steel slag–desulfurization gypsum ternary system and a quaternary system incorporating MSWI fly ash were established to compare their mechanical performance and reaction characteristics under different activation conditions. Furthermore, a multi-objective optimization strategy was developed by simultaneously considering compressive strength, flowability, and setting time in order to identify mix proportions suitable for engineering application. Finally, the evolution of reaction products, the construction of the load-bearing skeleton, and the reconstruction of pore structure were elucidated by means of isothermal calorimetry, XRD, SEM, and MIP, together with an evaluation of leaching safety. This study is expected to provide both theoretical support and technical guidance for the synergistic high-value utilization of low-titanium slag and MSWI fly ash in underground cemented paste backfill.

## 2. Materials and Methods

### 2.1. Materials

Five representative industrial solid wastes were employed as raw materials in this study, including ground low-titanium slag (LTS), steel slag (SS), and ground flue gas desulfurization gypsum (FGDG), all obtained from Shuicheng Iron and Steel Company, Guizhou, China; MSWI fly ash collected from a municipal solid waste incineration power plant in Guizhou, China; and lead–zinc tailings sourced from Xiangrong Mining Company, Puding County, Guizhou, China. Prior to use, LTS, SS, and FGDG were milled into fine powders to achieve particle sizes and specific surface areas compatible with binder reactions. All raw materials were dried and stored in sealed containers to minimize the influence of moisture fluctuations on mix proportioning and reaction kinetics.

The chemical compositions of the raw materials are summarized in Table 1. LTS exhibits a typical Ca-Si-Al reactive building-block chemistry, providing essential network-forming units for the construction of aluminosilicate cementitious phases and thus offering a material basis as a potentially reactive slag precursor. The relatively high CaO contents in MSWI fly ash and SS are expected to promote early-stage Ca-bearing phase dissolution and release of Ca^2+^, thereby increasing the effective activity of calcium ions in the pore solution. This, in turn, can drive LTS dissolution and facilitate subsequent precipitation of hydration products. FGDG is dominated by CaO and SO_3_, serving as an external sulfate source to regulate the reaction pathway.

**Figure 1:**
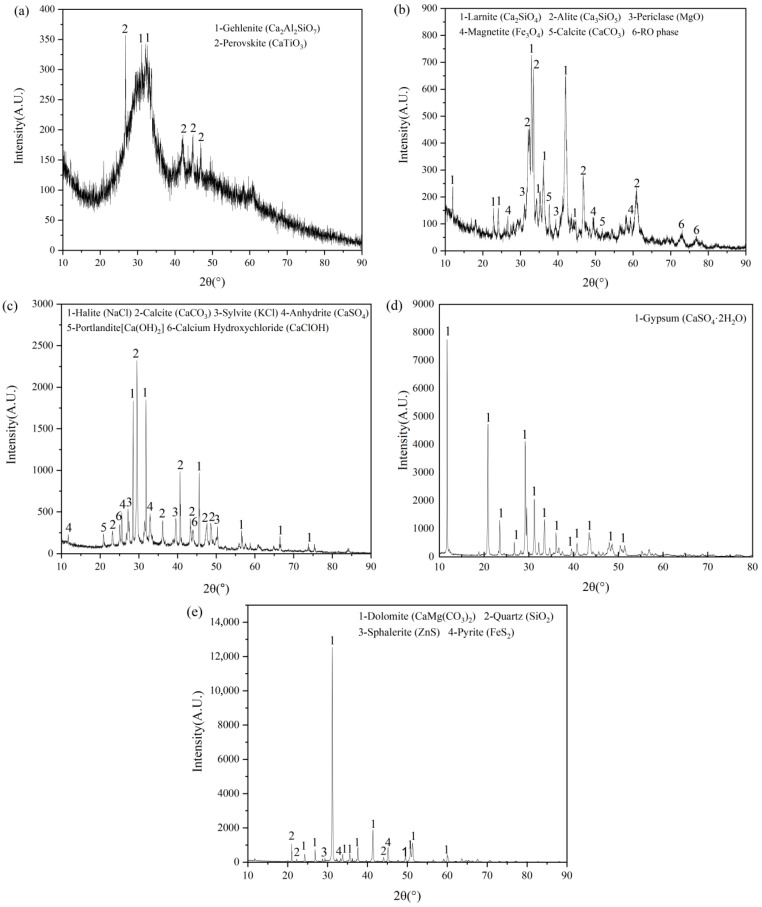
The XRD patterns of the raw materials ((**a**): LTS; (**b**): MSWI fly ash; (**c**): steel slag; (**d**): FGDG; (**e**): tailings).

The XRD patterns of LTS, SS, MSWI fly ash, FGDG, and lead–zinc tailings are presented in Figure 1. LTS shows a mineral assemblage broadly analogous to that of conventional slags, with a glassy phase as the dominant component, suggesting appreciable latent hydraulic reactivity, and minor crystalline phases, including gehlenite and perovskite (Figure 1a). MSWI fly ash is dominated by soluble chlorides with minor Ca-bearing phases (Figure 1b), which can rapidly increase pore solution alkalinity and thereby accelerate LTS depolymerization. SS contains reactive silicates and Ca/Mg-bearing phases, imparting strong alkalinity and an alkaline-activation effect (Figure 1c). FGDG is dominated by gypsum (CaSO_4_·2H_2_O) (Figure 1d), enabling a steady sulfate supply and regulating the formation and evolution of sulfate-bearing hydration products. Tailings consist mainly of dolomite, accompanied by minor sphalerite, quartz, and other mineral phases (Figure 1e).

**Figure 2:**
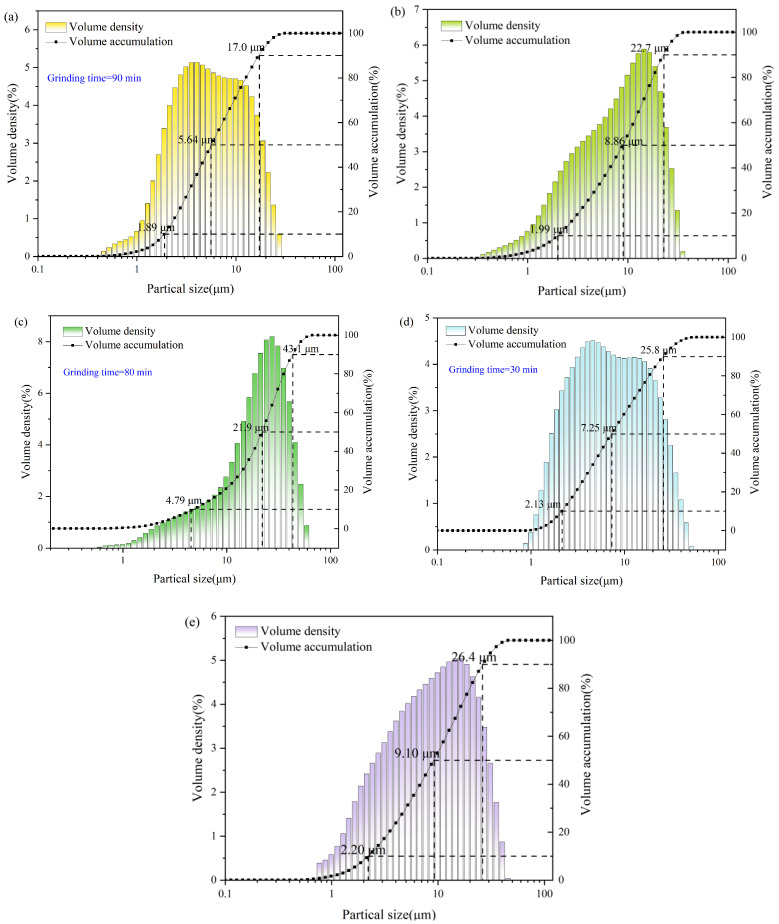
The particle size distributions of the five raw materials: (**a**) milled LTS, (**b**) non-milled MSWI fly ash, (**c**) milled SS, (**d**) milled FGDG, and (**e**) non-milled lead–zinc tailings.

The particle size distributions of all five raw materials were determined by laser diffraction to ensure controllable particle packing and reaction kinetics (Figure 2). After milling, LTS, SS, and FGDG exhibit predominantly micron-scale distributions; notably, LTS shows a D50 of 5.66 μm (Figure 2a), which promotes rapid dissolution–precipitation, gel growth, and densification via improved particle packing. By contrast, MSWI fly ash is relatively coarser (Figure 2b), and is therefore expected to contribute mainly through early-stage ionic release and skeletal filling. Tailings display a D50 of 9.10 μm (Figure 2e), complementing the fine powders to optimize overall gradation and enhance slurry structural stability. MSWI fly ash and lead–zinc tailings were used as received without any milling treatment (Figure 2b,e).

### 2.2. Method

#### 2.2.1. Reactivity Testing of Low-Titanium Slag

To quantify how pozzolanic reactivity varies with fineness, low-titanium slag (LTS) was ground for different durations to obtain a graded series of fineness levels. The milling times were 50, 70, 90, 110, and 130 min, as specified in Table 2. Immediately after grinding, the powders were sealed and stored to prevent moisture uptake, which could otherwise bias fineness control and subsequent reaction behavior. LTS reactivity was evaluated using the mortar strength contribution approach. Mortar mix proportions followed those outlined in Table 2; specimen preparation and compressive strength testing were performed in accordance with GB/T 17671-2021 [27]. The activity index was calculated at 3, 7, and 28 d following GB/T 18046-2017 [28].

#### 2.2.2. Specimen Preparation

Two categories of specimens were prepared: binder specimens and cemented backfill specimens. The binder system comprised an LTS-based ternary formulation and a quaternary formulation incorporating MSWI fly ash; the corresponding mix designs are provided in Table 3 and Table 4, respectively. The water-to-binder ratio was fixed at 0.32. Mix proportions for cemented backfill materials are summarized in Table 5, where different operating conditions were established by varying the solid concentration and the mortar ratio.

Binder specimens were cast in 30 × 30 × 50 mm molds, whereas mortar specimens were cast in 40 × 40 × 160 mm molds. For each mixture, constituents were weighed according to the designed proportions and mixed using mechanical stirring. After casting, specimens were cured at 20 °C and 98% relative humidity; they were demolded after approximately 24 h and then returned to the same curing environment until testing. Mechanical performance was evaluated at 3, 7, 28, and 90 d. Three replicate specimens were prepared for each condition to enable statistical analysis.

#### 2.2.3. Working Performance of Backfill Slurry and Leaching Toxicity Test

Working performance of the backfill slurry were characterized by flowability and setting time. Flowability was determined following Chinese standard GB/T 2419-2005 [29], and setting time was measured using a Vicat apparatus in accordance with Chinese standard GB/T 1346-2024 [30]. Leaching toxicity was evaluated following the horizontal oscillation extraction procedure specified in the Chinese standard HJ 557-2010 [31] for solid wastes. At the designated curing age, hardened backfill specimens were crushed and sieved to <3 mm, followed by extraction at the prescribed liquid-to-solid ratio. The leachates were filtered through 0.45 μm membranes prior to heavy metal concentration determination.

#### 2.2.4. A Multi-Objective Decision Model for Optimizing Backfill Materials Mix Proportions

A hybrid framework integrating AHP-based weighting, TOPSIS ranking, and RSM sweet spot modeling was established to determine an optimal backfill mix that balances three competing engineering requirements, namely compressive strength C1, flowability C2, and final setting time C3.

(1) AHP Weighting Determination

AHP was employed to quantify the relative importance of the three criteria. A pairwise comparison matrix A was constructed following the safety first principle and the weight vector W was obtained using the geometric mean method, together with a consistency check. The matrix A was set as in Equation (1).

(1) $$A=\left[\begin{array}{ccc} 1 & 3 & 3\\ 1/3 & 1 & 1\\ 1/3 & 1 & 1 \end{array}\right]$$

The maximum eigenvalue was λ_max_ = 3.00 and the consistency ratio was CR = 0.00, satisfying the consistency requirement CR < 0.10. The final weights were W = [0.60, 0.20, 0.20] for C1, C2, and C3.

(2) Ranking via TOPSIS

TOPSIS was applied to rank the valid mix groups. The experimental results were first organized into a decision matrix and normalized to remove dimensional differences (Equation (2)). Using the AHP-derived weights, a weighted normalized matrix was constructed, and the Positive Ideal Solution (A^+^) and Negative Ideal Solution (A^−^) were defined according to the benefit and cost attributes of the criteria. The Euclidean distances from each mix to A^+^ and A^−^ were then calculated to quantify its proximity to the ideal solutions (Equation (3)), which was subsequently used for overall ranking.

(2) $$z_{\mathrm{ij}}=\frac{x_{ij}}{\sqrt{\sum_{i=1}^{m}x_{ij}^{2}}}$$

(3) $$D_{i}^{+}=\sqrt{\sum_{j=1}^{n}\omega_{j}^{2}{\left(z_{ij}-z_{j}^{+}\right)}^{2}},\;D_{i}^{\_}=\sqrt{\sum_{j=1}^{n}\omega_{j}^{2}{\left(z_{ij}-z_{j}^{\_}\right)}^{2}}$$

(3) RSM sweet spot modeling and robustness verification

To obtain a robust feasible region beyond discrete experimental points, RSM was further applied to fit second-order polynomial models and identify a sweet spot under engineering constraints. The feasible domain was defined as Equation (4):

S = {(x1, x2) | Y_UCS_ ≥ 4 MPa} ∩ {Y_Flow_ ≥ 150 mm} ∩ {Y_final setting time_ ≤ 30 h} (4)

where x1 and x2 are the design variables. The sweet spot region within S was used to recommend an optimal mix, which was subsequently verified by confirmation experiments, with the TOPSIS optimal group serving as a reference.

### 2.3. Micro-Analysis of Paste

After curing to the designated ages, hydration was terminated using anhydrous ethanol, followed by crushing and vacuum drying prior to microstructural characterization. XRD were identified by a Rigaku DMAX RB12KW rotary anode X-ray diffractometer (Rigaku Corporation, Tokyo, Japan) with Cu Kα radiation, operated over 3° to 90° with a step size of 0.02° and a scanning rate of 8° per minute. The heat of hydration was continuously recorded using a TAM Air eight channel isothermal microcalorimeter (TA Instruments, New Castle, DE, USA) at 20 °C. Micromorphologies were examined using an FEI Quanta 650 scanning electron microscope (FEI Company, Hillsboro, OR, USA) at an accelerating voltage of 10 kV, with spot size 6 and aperture 3. Pore structure was measured by mercury intrusion porosimetry using an AutoPore IV 9500 V1.09 instrument(Micromeritics Instrument Corporation, Norcross, GA, USA), from which the cumulative intrusion curve and pore size distribution were obtained.

## 3. Results

### 3.1. Assessment of the Latent Pozzolanic Reactivity of Low-Titanium Slag

**Figure 3:**
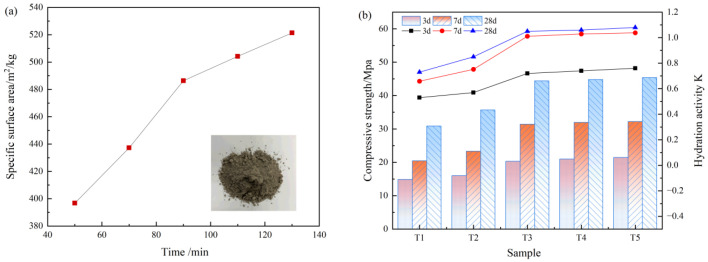
Results of the reactivity test for low-titanium slag ((**a**): specific surface area of low-titanium slag as a function of grinding time; (**b**): comparison of compressive strength and pozzolanic activity of low-titanium slag mortar specimens at different curing ages).

As shown in Figure 3, increasing the grinding time enhances both compressive strength and the activity index at all curing ages, confirming that mechanical comminution effectively activates the pozzolanic reactivity of low-titanium slag. The largest performance gain occurs at 90 min, beyond which further grinding provides only marginal improvement, indicating a clear critical fineness for activation that is consistent at 3 d, 7 d, and 28 d. Considering the balance between reactivity enhancement and grinding energy and cost, 90 min was selected as the optimal condition. Under this condition, the 28 d activity index reaches 108% and satisfies the S95 requirement at 28 d.

### 3.2. Preparation of Low-Titanium-Slag-Containing Cementitious Materials

#### 3.2.1. Effect of Steel Slag on the Activation of Low-Titanium-Slag-Based Cementitious Materials

**Figure 4:**
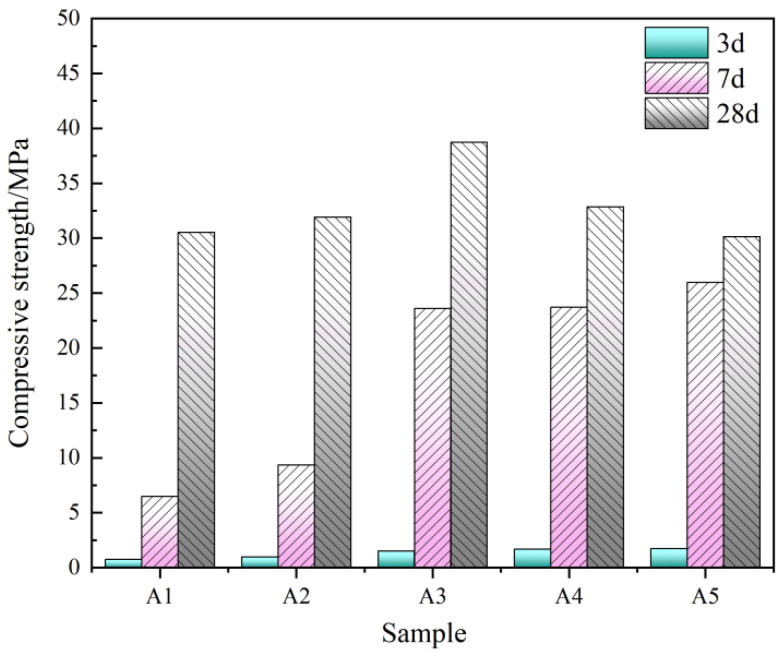
Compressive strength of low-titanium-slag-based ternary cementitious system.

As shown in Figure 4, the low-titanium slag–steel slag–FGDG ternary binder shows continuous compressive strength development from 3 to 28d, indicating that compressive strength gain is governed by mid-to-late stage reactions and progressive densification. The compressive strength of A3 sample at 28 d exhibits a clear threshold-type response, reaching a maximum at 43% low-titanium slag and decreasing at higher contents, which defines a distinct optimum window. This behavior points to an intrinsic limitation of the ternary system: the slow hydrolysis of steel slag provides insufficient early-age alkalinity and reactive ions to sustain activation of the low-titanium slag glass phase; at higher dosages, partially reacted residues reduce packing efficiency and weaken late-stage densification. Accordingly, introducing an additional component that can rapidly intensify early-age activation is necessary to increase the effective reactivity of low-titanium slag and broaden the controllable mix design space.

#### 3.2.2. Effect of MSWI Fly Ash on the Activation of Low-Titanium-Slag-Based Cementitious Materials

**Figure 5:**
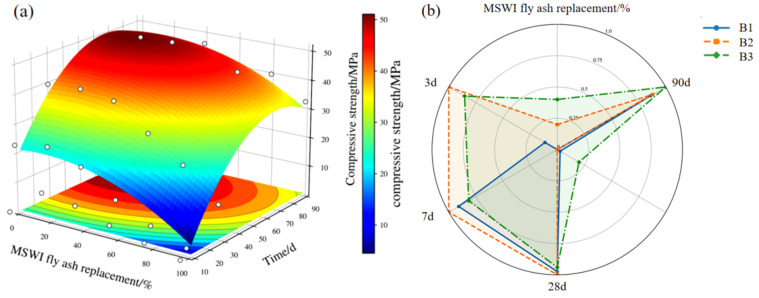
The results of compressive strength of quaternary binder system ((**a**): the response surface fitted to the measured compressive strength; (**b**): the radar plot of compressive strength).

On the basis of the optimal ternary proportioning scheme, MSWI fly ash was introduced to develop a quaternary binder system comprising low-titanium slag, steel slag, FGDG, and MSWI fly ash, thereby intensifying the activation of the low-titanium slag. As shown in Figure 5a, the response surface fitted to the measured compressive strength data demonstrates a clear age-dependent sensitivity of strength to the MSWI fly ash replacement ratio in the quaternary binder. At early ages, the high-strength region is concentrated at low-to-moderate replacement levels, implying more efficient reaction initiation and faster establishment of an initial load-bearing framework; correspondingly, B2 exhibits the most pronounced and responsive strength gains at 3 and 7 d. With increasing curing age, the high-strength region shifts toward higher replacement ratios, indicating that mid-to-late stage reactions and microstructural densification are better supported by a moderately higher fly ash contribution, consistent with the superior performance of B3 at 28 and 90 d.

This evolution is further confirmed by the radar plot in Figure 5b, where B2 presents the largest radii at 3 and 7 d, whereas B3 dominates at 28 and 90 d, evidencing a stronger long-term strength buildup. Collectively, the response surface mapping and radar-based cross-validation identify two distinct optimization windows within the quaternary design space, with B2 and B3 preferentially optimizing early-age and late-age performance, respectively; these formulations are therefore selected as representative candidates for subsequent backfill mix optimization and mechanistic interrogation.

**Figure 6:**
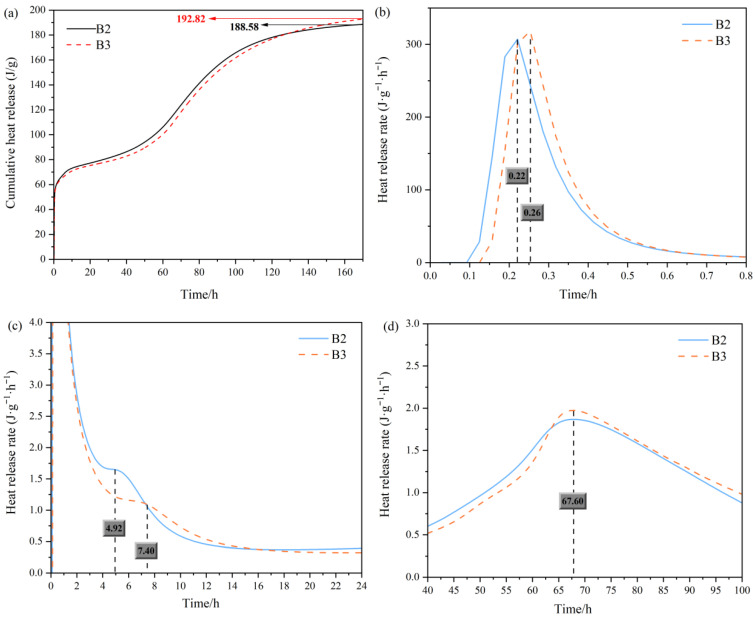
Hydration heat evolution of B2 and B3 mixtures. (**a**): cumulative heat; (**b**): heat release rate at initial period; (**c**): heat release rate at 24 h; (**d**): heat release rate in the late stage.

As shown in Figure 6, the heat of hydration of B2 and B3 is characterized by a rapid early exothermic burst followed by a low-rate yet sustained heat release regime, with the contrast between the two mixtures indicating a trade-off between reaction onset and long-term reaction extent. During the initial period (0.1–0.8 h), both systems display a pronounced first peak, reflecting rapid dissolution coupled with early nucleation–precipitation. Notably, the main heat-flow peak of B3 is slightly delayed relative to B2, with the peak time shifting from 0.22 h (B2) to 0.26 h (B3), suggesting a more tempered early-stage onset in B3 (Figure 6b). In the late stage (60–160 h), a third peak becomes evident; although the instantaneous heat release rate is modest, its extended persistence signifies continued reactions and secondary product formation that progressively densify the matrix. Both mixes show a broad feature centered at 67.60 h (Figure 6d), yet B3 remains marginally higher and more persistent, yielding a slightly greater cumulative heat of hydration (192.82 J·g^−1^) than B2 (188.58 J·g^−1^) (Figure 6a). Overall, the heat of hydration results suggest that B2 prioritizes rapid early reactivity, whereas B3 attains a higher overall reaction extent with a stronger late-stage kinetic drive, providing a more robust basis for sustained long-term strength development and supporting the selection of B3 as the preferred formulation for subsequent validation.

### 3.3. Preparation of Low-Titanium-Slag-Containing Cemented Backfill Materials

#### 3.3.1. The Workability of Low-Titanium-Slag-Containing Cemented Backfill Materials

**Figure 7:**
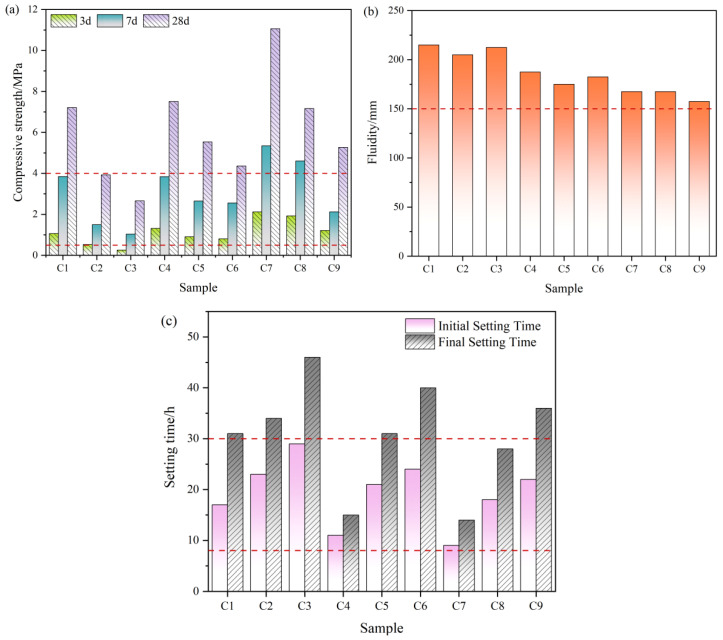
The workability characterization of solid-waste-derived cemented backfill materials ((**a**): compressive strengths; (**b**): fluidity; (**c**): setting times).

Figure 7 summarizes the measured fluidity, setting times, and compressive strengths for solid-waste-derived cemented backfill materials incorporating low-titanium slag, jointly describing the key performance during placement and in-service stages.

All samples exhibit fluidity values of 157.5–215 mm when used as underground cemented backfill materials (Figure 7a), all samples exceeding the 150 mm pipeline transport criterion, thereby indicating baseline conveyability and pumpability across the formulation matrix. The high-solids samples (C7–C9) approach this lower bound, implying increased operational sensitivity to minor fluctuations in water content and to variations in pipeline hydraulic resistance.

For setting behavior, the initial setting times span 9~29 h, consistently satisfying the workability requirement of an initial setting time exceeding 8 h. The final setting times are comparatively prolonged, indicating slower formation of the load-bearing skeleton and delayed hardening in this binder system.

In terms of compressive strength, the C7 and C8 samples exceed 4 MPa at 7 d, supporting applications requiring high early-age load-bearing capacity, such as backfill for artificial sill pillars. Most other samples remain below 4 MPa at 7 d, indicating limited early bearing capacity and a compressive strength contribution governed primarily by sustained late-age hardening. At 28 d, C7 delivers the highest compressive strength among all samples (11.07 MPa). C3 sample attains only 2.66 MPa at 28 d, which is insufficient for load-bearing backfill; however, it meets the low-strength criterion for non-structural placement (>1 MPa) and is therefore better suited to scenarios where densification and void filling dominate functional requirements.

#### 3.3.2. Formulation Optimization of Low-Titanium-Slag-Containing Cemented Backfill Materials Using a Multi-Objective Decision Model

**Figure 8:**
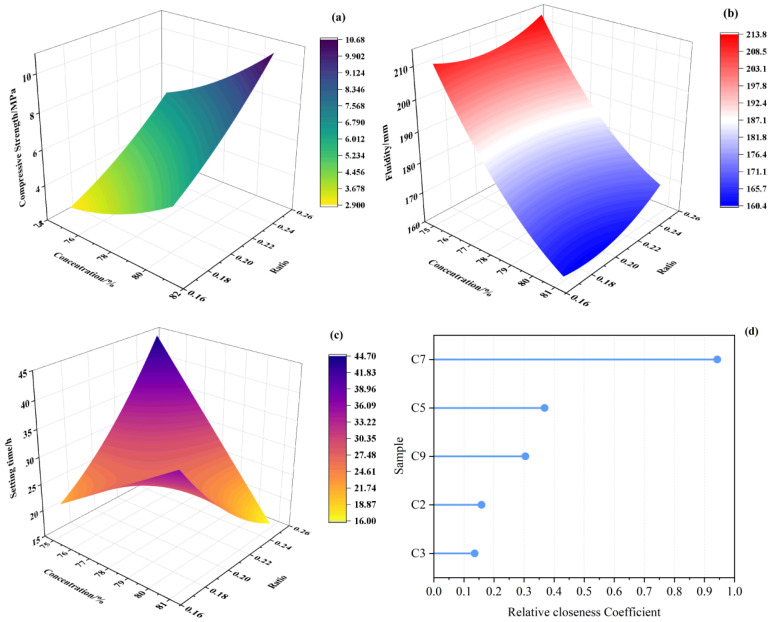
The 3D response surfaces of workability characteristics and TOPSIS-based ranking of all eligible mix proportions. (**a**): compressive strengths; (**b**): fluidity; (**c**): setting times; (**d**): TOPSIS ranking.

The 3D response surface plots visually decouple the interactive effects of experimental variables (Figure 8). A steep ascent surface is observed in the high-concentration/high-ratio region (Figure 8a). The strength increases exponentially as the solid content rises from 75% to 81%, confirming that porosity reduction is the primary strengthening mechanism. Contrastingly, flowability shows a negative correlation with solid content. The surface gradient flattens at higher concentrations, indicating that the optimized particle size distribution of the tailings helps mitigate viscosity buildup even at 81% concentration (Figure 8b). Figure 8d illustrates the TOPSIS ranking of all eligible mix proportions. The calculation results reveal a clear hierarchy in comprehensive performance. With a slurry concentration of 81% and a binder-to-tailings ratio of 1:4, the C7 sample achieved the highest relative closeness coefficient (C_i_ = 0.943). It exhibits a compressive strength of 11.07 MPa at 28 d, a fluidity of 167.5 mm, and a final setting time of 14 h. Its dominance is attributed to the minimal distance to the Positive Ideal Solution (D_i_^+^ = 0.021). The selection of the optimal mix represents a strategic trade-off between rheological behavior and mechanical development. While traditional high-concentration slurries often suffer from poor transportability, the C7 sample maintains a flow spread of 167.5 mm. This phenomenon can be explained by the “Lubrication Layer Theory”—the excessive binder paste at the 1:4 ratio acts as a lubricant coating the tailings particles, effectively reducing the yield stress despite the high solid volume fraction.

**Figure 9:**
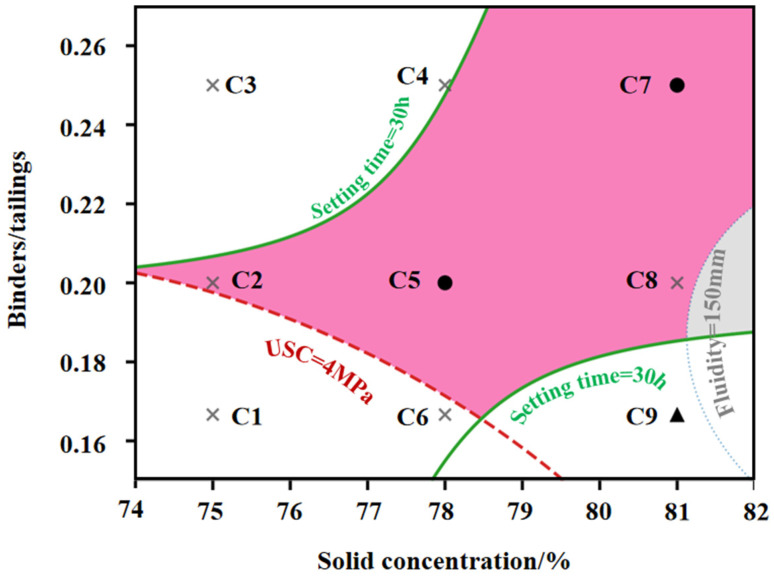
The sweet spot analysis of the workability characterization of solid-waste-derived cemented backfill materials.

A critical contribution of this study is the validation of solution robustness via the sweet spot analysis (Figure 9). The pink-shaded region represents the feasible operation domain satisfying all strict constraints (UCS ≥ 4 MPa, fluidity ≥ 150 mm, final setting time < 30 h). The optimal point the C7 sample is located deeply within this domain rather than on the boundary lines. This spatial positioning implies a high safety margin. Even if minor fluctuations in slurry concentration or binder dosage occur during large-scale industrial preparation, the material performance is predicted to remain within the “Sweet Spot”, thereby reducing the likelihood of pipe blockage arising from inadequate flow and structural failure resulting from insufficient strength.

Based on the AHP-TOPSIS ranking and RSM verification, the C7 sample is identified as the optimal backfill materials, offering a robust balance of high strength (11.07 MPa), pumpable fluidity (167.5 mm), and efficient setting time (14 h).

#### 3.3.3. Leaching Toxicity Characteristics of Hazardous Constituents in Low-Titanium-Slag-Containing Cemented Backfill Materials

**Figure 10:**
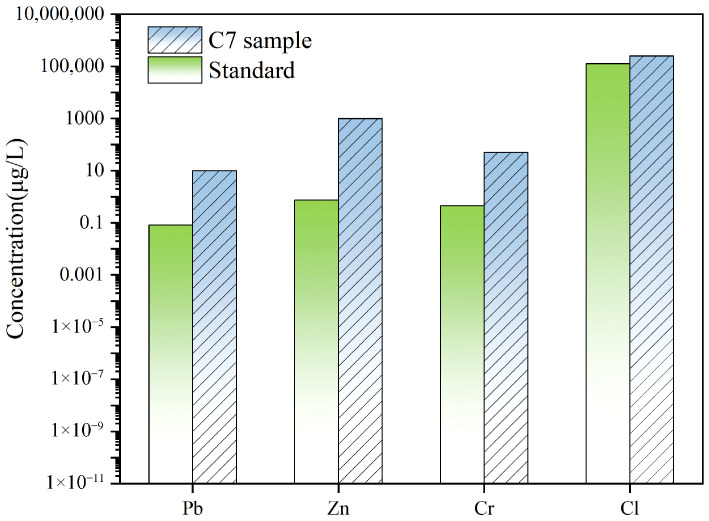
The leaching concentration of heavy metals in backfill materials.

Figure 10 compares the leaching concentrations of heavy metals and soluble Cl from the C7 cemented backfill materials with the Class III limit values prescribed in China’s Groundwater Quality Standard (GB/T 14848-2017 [32]). The leached concentrations of Pb, Zn, and Cr are uniformly low and remain below the corresponding Class III thresholds, evidencing effective solidification/stabilization of these metals within the backfill matrix and indicating a low leaching risks. In addition, the concentration of soluble Cl is maintained below the Class III criterion, demonstrating suppression of soluble-anion mobilization and compliance with the groundwater environmental admissibility requirements. Collectively, the compliance-based assessment supports the leaching safety and engineering applicability of the C7 matrix for underground cemented backfilling.

### 3.4. Microstructural Mechanisms Governing Compressive Strength Development in Low-Titanium-Slag-Containing Cemented Backfill Materials

#### 3.4.1. MIP Analysis

Porosity is a primary determinant of the performance of construction materials, particularly porous media. For cemented backfill, pore structures inevitably develop within the hardened body as a consequence of water loss during curing, as well as the degree of interfacial densification and bonding between hydration products and aggregate particles. Such pore development can, in turn, induce variability in the compressive strength of the consolidated backfill. Accordingly, this paper employs mercury intrusion porosimetry (MIP) to systematically characterize the evolution of the pore structure of the C7 matrix at different hydration ages and to elucidate its implications for the compressive strength of the backfill specimens.

In general, pores are commonly classified into three categories according to pore diameter: small gel pores (3.5–10 nm), large gel pores (10–100 nm), and capillary pores (>100 nm). In concrete, the pore system is further categorized in terms of its relevance to durability and mechanical performance as harmless pores (<4.5 nm, typically referring to intragel micropores within C-(A)-S-H), less harmful pores (4.5–50 nm), harmful pores (50–100 nm), and highly harmful pores (>100 nm). Macroporosity is mainly associated with entrapped, closed air voids, whereas microporosity is primarily attributed to capillary and gel pores formed during hydration.

**Figure 11:**
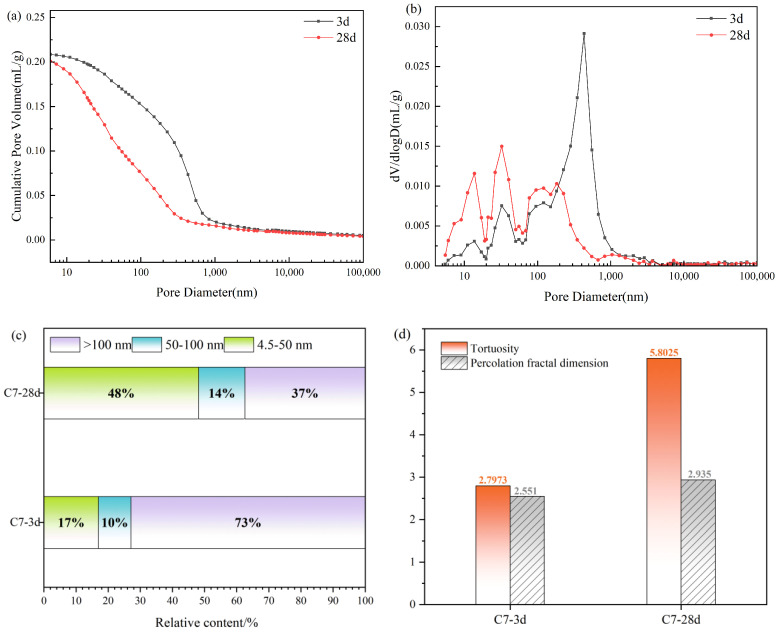
The pore size distribution of C7 sample at different curing times. (**a**): cumulative pore volume distribution; (**b**): differential pore size distribution; (**c**): relative proportion of pores in different size ranges; (**d**): comparison of tortuosity and percolation fractal dimension.

Figure 11 presents the cumulative intruded pore volume and differential pore size distribution of the C7 backfill materials at hydration ages of 3 d and 28 d. As shown in Figure 11a, pronounced pore structure densification occurs during the 3–28 d hydration period, the cumulative pore volume curve shifts leftward overall, with the most evident reduction within the 10–1000 nm range, indicating sustained consumption of pore volume dominated by capillary pores. Concurrently, the pore size distribution evolves from a sharp dominant peak centered at 400 nm at 3 d to a markedly attenuated main peak that migrates toward 10–50 nm and develops a multi-peak profile at 28 d (Figure 11b). This transition reflects progressive filling of pore channels by hydration products, accompanied by pore-throat constriction and refinement, as well as a concomitant reduction in connectivity.

The hierarchical statistics further corroborate this evolution. The fraction of highly harmful pores (>100 nm) decreases substantially from 73% to 37%, whereas gel pores in the 4.5–50 nm range increase from 17% to 48% (Figure 11c). The proportion of harmful pores (50–100 nm) remains comparatively stable at 10–14%. These results indicate that pore structure refinement is governed primarily by the transformation of large pores into medium-to-fine pores rather than by a uniform, proportional shrinkage across the entire pore spectrum. In parallel, the increase in tortuosity from 2.80 to 5.80 (Figure 11d) indicates that the internal pore network became more winding and less connected, which substantially increased the mass-transfer resistance within the matrix. As a result, the inward penetration of the leaching medium and the outward diffusion of dissolved heavy metal species were both hindered. Combined with the refinement of pore structure and the reduction in harmful pores, this structural evolution contributed to a denser transport barrier, thereby suppressing the leaching of Pb, Zn, and Cr. Therefore, the heavy metal leaching results shown in Figure 10 can be partly attributed to the increased tortuosity and the corresponding restriction of contaminant migration pathways. Collectively, the reduced pore connectivity and enhanced matrix densification reinforce the load-bearing skeleton of the hardened backfill materials.

#### 3.4.2. SEM Analysis

**Figure 12:**
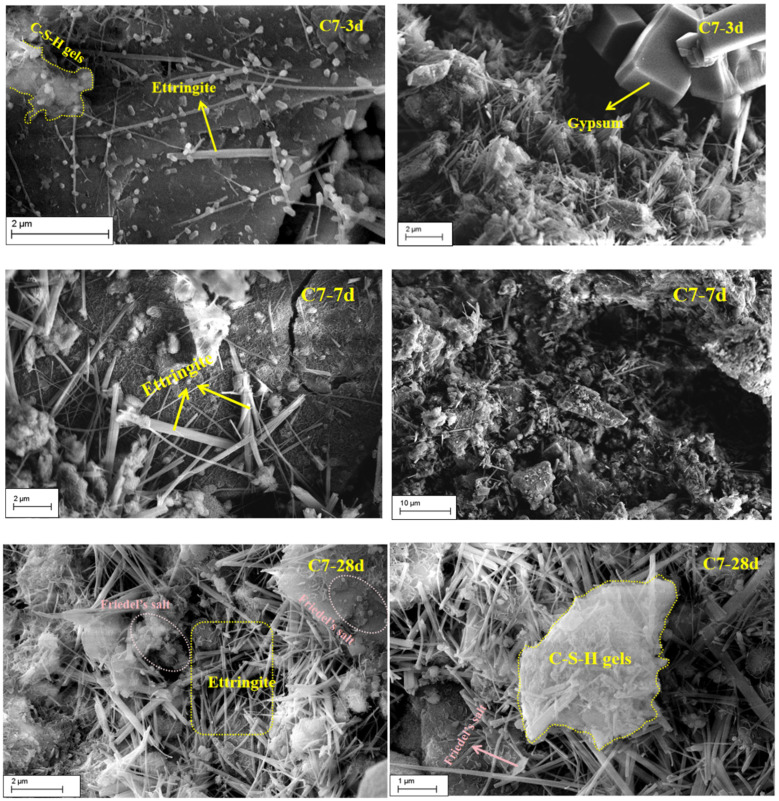
The SEM results of the C7 sample at different curing times.

Figure 12 presents SEM micrographs of the C7 backfill materials at hydration ages of 3, 7, and 28 d, delineating hydration product evolution and its contribution to matrix densification and compressive strength development. Only a limited amount of short and acicular ettringite is observed at 3 d, with crystals sparsely distributed and the C-S-H gel phase remaining weakly developed; unreacted low-titanium slag particles coexist with an open pore network, indicating inadequate interfacial cohesion. An extensive precipitation of ettringite is observed at 7 d, characterized by intergrown prismatic-to-columnar crystals (1.5–2.5 μm) that interlace into a networked framework, while concomitantly formed gel products infill residual voids and encapsulate the crystalline phase to generate composite agglomerates; a distinct reaction transition layer (0.5–1 μm) becomes evident at the aggregate–binder interface, accompanied by a pronounced reduction in interfacial porosity. A continuous three-dimensional load-bearing skeleton is observed at 28 d, arising from further coarsening of ettringite (~3–6 μm) and its intimate interweaving with the gel phase; strengthened encapsulation and pore-filling effects promote progressive pore refinement and closure, thereby advancing microstructural densification and sustaining gains in compressive strength.

## 4. Discussion

### 4.1. Activation and Assessment of Potential Hydration Activity in Low-Titanium Slag

The intrinsic characteristics of low-titanium slag indicate that it is better classified as an activatable latent cementitious precursor rather than a material with inherently high, self-sustaining hydration reactivity. Its chemical composition is dominated by CaO, accompanied by relatively high contents of SiO_2_ and Al_2_O_3_ (Table 1), which provides the essential Ca–Si–Al building units for the subsequent formation of Ca–Si–Al–type cementitious phases and sulfoaluminate phases. Meanwhile, XRD reveals the coexistence of a pronounced amorphous hump with crystalline phases such as gehlenite and perovskite (Figure 1a), implying that low-titanium slag possesses substantial latent hydraulic potential. However, this potential does not spontaneously translate into an effective reaction contribution. Instead, it is constrained by the multiphase architecture, where reactive fractions are physically encapsulated and their dissolution is kinetically hindered. Consequently, the reactivity of low-titanium slag must be unlocked through targeted activation strategies. The results of physical activation provide direct evidence: after grinding increased the specific surface area to 486 m^2^/kg, the activity index surpassed the critical threshold and remained above 1 at 28 d (Figure 3), indicating a functional transition from weak reactivity to a meaningful contribution to hydration once a key fineness is achieved. Accordingly, the low-titanium slag ground for 90 min to reach 486 m^2^/kg was adopted for the preparation of the subsequent cementitious materials in this study.

Moreover, calorimetric data further corroborate—on a reaction extent basis—the sustained development of this latent reactivity under chemical activation. At a constant low-titanium slag content, the system activated solely by steel slag exhibited a 7 d cumulative heat release of 188.58 J/g, whereas substituting 40% of the steel slag with MSWI fly ash increased the value to 192.82 J/g (Figure 6). This enhancement indicates that synergistic activation can markedly increase the early cumulative reaction output, thereby enabling more complete participation of the reactive fractions in low-titanium slag.

These results indicate that low-titanium slag is an activatable latent hydraulic precursor, the reactivity of which is jointly controlled by grinding-induced interfacial activation and the highly reactive regime established by alkali activation. Their synergy determines the dissolution behavior of low-titanium slag and the efficiency of subsequent product evolution. Moreover, the alkalinity and Ca supplied by steel slag, together with the reactive Al species and soluble salts introduced by MSWI fly ash, further reinforce the reaction environment, thereby enabling the latent reactivity of low-titanium slag to be systematically exploited.

### 4.2. Feasibility of Preparing Low-Carbon Cementitious Materials from Low-Titanium Slag

The transformation of low-titanium slag from a multiphase solid waste with low reaction efficiency into an engineering-viable cementitious precursor depends primarily on whether mix design can effectively overcome the constraints on its dissolution and subsequent reaction conversion, rather than on simply increasing the dosage of individual components. The strength results of the ternary system indicate that, when the contents of low-titanium slag and desulfurization gypsum were fixed at 43% and 14%, respectively, the system possessed the essential Ca-Si-Al reactive units and a sustained supply of SO_4_^2−^ (Table 3). Nevertheless, when the remaining activation was provided solely by steel slag, the latent reactivity of low-titanium slag was still insufficiently mobilized. The compressive strength of A3 was only 39.15 MPa at 28d, indicating that although steel slag supplied sufficient alkalinity and Ca to sustain a certain degree of reaction, single-source activation remained inadequate to support both early ionic release and the continued formation of reaction products in a precursor with inherently low reactivity such as low-titanium slag (Figure 5). Therefore, the limited performance of the ternary system was governed not by an insufficient reserve of framework components, but by the restricted capacity of the activator system to establish a sufficiently favorable reaction environment.

The advantage of the quaternary system lies in its targeted mitigation of this limitation. With the contents of low-titanium slag and desulfurization gypsum unchanged, the incorporation of MSWI fly ash transformed the activator system from steel-slag-only activation into steel slag–fly ash composite activation, thereby markedly improving the reaction environment. In this system, steel slag mainly provided alkalinity and Ca support, whereas MSWI fly ash contributed reactive Al species and soluble salt components. Their combination produced a complementary effect between early ion-supply efficiency and the maintenance of a favorable alkaline environment at later stages, thereby enhancing both the dissolution of reactive components from low-titanium slag and the subsequent conversion of these species into effective cementitious products. This result suggests that the role of MSWI fly ash was not limited to increasing the overall reaction extent, but was more critically associated with improving the efficiency of strength-contributing product formation within a given curing period. In other words, composite activation promoted a more complete transformation of low-titanium slag from a latent reactive precursor into an effective source of cementitious phases.

The material-level improvement achieved by composite activation was further reflected in the engineering adaptability of the underground cemented backfill system. When B3 was used as the binder for backfill preparation, the performance variations among C1–C9 demonstrated that the evaluation of low-titanium-slag-based systems should not be confined to the intrinsic strength of the binder, but should also account for the coupled relationship among strength, flowability, and setting time under practical backfilling conditions. Increasing slurry concentration and decreasing the binder-to-tailings ratio enhanced the load-bearing capacity of the backfill, but generally at the expense of flowability and with corresponding changes in setting behavior. This indicates that the optimization of underground backfill materials is inherently a multi-objective problem, for which a single performance indicator is insufficient to represent engineering applicability. Accordingly, a multi-criteria decision analysis was adopted to assess strength, flowability, and setting performance within a unified framework, thereby converting multiple response variables into a comparable and reproducible route for mix proportion optimization. The results showed that, for load-bearing applications, the C7 mixture, characterized by high slurry concentration and a low binder-to-tailings ratio, exhibited the best overall performance, with 7 d strength exceeding 4 MPa and 28d strength remaining within the upper range of the system. By contrast, for non-load-bearing backfill applications, greater priority should be given to construction flowability and operational time window, and mixtures with higher flowability and more suitable setting characteristics should be preferred provided that the compressive strength exceeds 1 MPa.

Overall, the present results demonstrate that the low reaction efficiency of low-titanium slag is not an intrinsic and insurmountable limitation, but rather a challenge that can be effectively addressed through composite activation design. Steel slag–fly ash composite activation enhanced the reaction utilization of low-titanium slag, enabling its more efficient conversion into cementitious products with substantive contributions to strength development. Furthermore, multi-criteria decision analysis established a practical link between material-level reaction advantages and the engineering requirements of underground cemented backfill, thereby providing an operable route for the evaluation and optimization of low-titanium-slag-based cementitious systems. These findings confirm that low-titanium slag can serve not only as a latent cementitious precursor in multi-solid-waste systems, but also as a promising binder source for underground cemented backfill materials.

### 4.3. Hydration Reaction Mechanism Analysis

The strength development of cemented backfill materials is fundamentally controlled by the ability of effective reaction products to rapidly establish a continuous load-bearing skeleton within the service-relevant curing period, while concurrently reducing the connectivity of the pore network. Because cumulative heat release is generally well correlated with both reaction extent and early-age strength development in cementitious systems, it can be used to distinguish differences in the effective reaction contribution among different mix formulations. In the present system, the quaternary mixture B3 exhibited a 7 d cumulative heat release of 192.82 J·g^−1^, compared with 188.58 J·g^−1^ for the ternary reference mixture B2, indicating that the incorporation of MSWI fly ash enhanced the effective extent of reaction from the early to intermediate stages. This observation is consistent with the microstructural evolution revealed by SEM. At 3 d, only a limited amount of short needle-like ettringite was observed, with the crystals remaining spatially isolated and failing to form an effective intergrown structure. By 7 d, ettringite had substantially increased in abundance and further developed into interwoven columnar crystals, progressively establishing an early spatial load-bearing framework. Meanwhile, a relatively continuous reaction rim formed within the interfacial regions of the particles, which contributed to reducing interfacial defects and improving overall matrix integrity. This evolution is consistent with the general behavior of sulfate-bearing systems, in which AFt preferentially precipitates and dominates early-stage structural build-up. These results therefore indicate that the rapid formation of ettringite and its interwoven spatial growth constituted a key source of early strength development in the present system. The MIP results further demonstrate that the enhancement in strength was governed primarily by pore network restructuring across multiple length scales, rather than by a simple reduction in total porosity. Taking the C7 matrix as an example, with increasing curing age, the proportion of harmful pores decreased from approximately 73% to approximately 37%, whereas the proportion of less harmful pores increased from approximately 17% to approximately 48%, indicating an overall shift in pore size distribution toward the finer pore range. At the same time, tortuosity increased from approximately 2.80 to approximately 5.80, and the fractal dimension rose from approximately 2.55 to approximately 2.94. These changes suggest that the internal transport pathways evolved from relatively direct connected channels into a more tortuous, heterogeneous, and topologically complex pore network, accompanied by a pronounced reduction in connectivity. Previous studies have shown that tortuosity and fractal parameters derived from MIP inversion can effectively characterize pore network connectivity and transport complexity. An increase in these parameters generally indicates greater obstruction of percolating pathways, reduced transport capacity, and enhanced overall structural stiffness.

Overall, the strength development of the present system can be attributed to the synergistic effects of ettringite-dominated early skeleton formation, progressive reinforcement of the interfacial transition zone, and the simultaneous refinement and depercolation of the pore structure. The increased heat release identified by isothermal calorimetry, the framework formation revealed by SEM, and the pore network evolution characterized by MIP corroborate one another across multiple scales, collectively supporting a coherent mechanistic interpretation of strength development in this system.

### 4.4. Limitations and Future Perspectives

Although the present study demonstrates, through mix design optimization and multiscale characterization, that low-titanium slag can be effectively activated under the synergistic action of steel slag, MSWI fly ash, and desulfurization gypsum, and that the resulting system can satisfy the engineering requirements of underground cemented backfill in terms of workability and strength grade, several key mechanisms remain insufficiently resolved. This limitation, to some extent, constrains a precise assessment of reaction controllability and the long-term environmental safety boundaries of the system. Accordingly, future work should focus primarily on the following two aspects.

From the perspective of reaction mechanisms, further efforts are needed to elucidate the factors governing the sustained high-level formation of ettringite within the engineering service period. Particular attention should be given to the temporal coupling between the effective release of Al sources under steel slag–fly ash composite activation and the continuous sulfate supply from gypsum. In addition, the specific roles of characteristic mineral phases in low-titanium slag, together with their interfacial regions, in heterogeneous nucleation, crystal inter-growth, and framework development should be identified, so as to establish a controllable mechanistic framework for the ettringite formation.

From the perspective of environmental safety, systematic investigation is still required to clarify the immobilization pathways, speciation-transformation routes, and long-term stability boundaries of heavy metals and soluble Cl originally associated with MSWI fly ash after the incorporation of low-titanium slag. Building on the present evidence for pore structure refinement and reduced connectivity, future studies should further discriminate the respective contributions of physical encapsulation and chemical fixation to contaminant stabilization. Particular emphasis should be placed on clarifying the coupled pathways involving solid-phase incorporation, interfacial adsorption, and encapsulation of heavy metals, as well as the solid-phase binding hosts, formation conditions, and potential remobilization risks of Cl. Such efforts will provide a verifiable mechanistic basis and corresponding control strategies for the coordinated optimization of strength development and environmental safety.

## 5. Conclusions

This study systematically evaluated the feasibility of using low-titanium slag as a major cementitious precursor in multi-solid-waste cemented paste backfill and clarified the roles of grinding activation, synergistic excitation, proportion optimization, and microstructural evolution in governing its engineering performance and environmental safety. The results demonstrate that low-titanium slag can be effectively activated under the combined action of steel slag, MSWI fly ash, and flue gas desulfurization gypsum, providing a feasible route for its high-value utilization in backfill materials.

(1) Mechanical grinding markedly improved the latent hydraulic reactivity of low-titanium slag. After 90 min of grinding, its 28 d activity index reached 108%, indicating that sufficient particle refinement is essential for transforming low-titanium slag into an effective cementitious precursor.
(2) In the low-titanium slag–steel slag–desulfurization gypsum ternary system, steel slag alone was insufficient to fully activate low-titanium slag, whereas the incorporation of MSWI fly ash significantly enhanced the synergistic activation effect. The quaternary system with 40% MSWI fly ash replacement exhibited the most favorable reaction extent and later-age strength development.
(3) Multi-objective optimization identified the optimum backfill proportion as a solid mass concentration of 81% and a binder-to-tailings ratio of 1:4. Under this condition, the material achieved satisfactory flowability and setting behavior, and the 28 d compressive strength reached 11.07 MPa.
(4) Compressive strength enhancement was primarily associated with the continuous formation of ettringite and gel phases, which progressively constructed a denser load-bearing skeleton and refined the pore structure from coarse-pore-dominated to fine-pore-dominated, thereby promoting matrix densification and mechanical development.
(5) The optimum formulation exhibited low leaching concentrations of Pb, Zn, Cr, and soluble Cl, all below the relevant groundwater quality limits, demonstrating favorable environmental safety alongside the efficient co-utilization of low-titanium slag and MSWI fly ash.

## Figures and Tables

**Table 1:** Chemical composition of raw materials.

Raw Materials	CaO	SiO_2_	Al_2_O_3_	TiO_2_	MgO	SO_3_	Fe_2_O_3_	MnO	Na_2_O	Cl	LOL
LTS	43.82	26.43	12.29	6.11	5.21	2.45	1.64	0.76	0.61	0.02	0.66
SS	45.03	11.76	1.35	3.41	4.43	1.79	26.11	4.43	0.03	0.04	1.62
MSWI fly ash	52.89	7.12	3.49	0.02	2.76	5.74	1.39	0.05	7.68	17.4	1.72
FGDG	52.77	1.79	0.34	0.22	0.26	43.12	0.42	0.l3	0.11	0.56	0.41
Tailings	25.99	48.80	3.99	0.04	10.56	3.62	4.67	0.29	0.16	0.47	1.41

Note: Low-titanium slag (LTS), steel slag (SS), ground flue gas desulfurization gypsum (FGDG).

**Table 2:** Experimental protocol for pozzolanic activity testing of low-titanium slag.

Sample	Grinding Time	Specific Surface Area	P·O 42.5 Portland Cement	LTS	Standard Sand	Water
	/min	/m^2^/kg	/g			
T0	/	/	450	/	1350	225
T1	50	396.73	225	225		
T2	70	437.24	225	225		
T3	90	486.35	225	225		
T4	110	504.19	225	225		
T5	130	521.34	225	225		

**Table 3:** Mix proportion design of low-titanium-slag-based cementitious system.

Sample	SS:LTS	SS	LTS	FGDG
	/	/wt. %		
A1	3:1	64.5	21.5	14
A2	2:1	57.3	28.7	14
A3	1:1	43	43	14
A4	1:2	28.7	57.3	14
A5	1:3	21.5	64.5	14

**Table 4:** Mix proportions design of MSWI fly ash and low-titanium-slag-based cementitious system.

Sample	MSWI Fly Ash Replacement Ratio	MSWI Fly	SS	LTS	FGDG
	/%	/wt. %			
B1	0	0	43	43	14
B2	20	8.6	34.4		
B3	40	17.2	25.8		
B4	60	25.8	17.2		
B5	80	34.4	8.6		
B6	100	43	0		

**Table 5:** Mix proportion design of cemented backfill materials.

Sample	Concentration	Binder-to-Sand Ratio	MSWI Fly Ash	SS	LTS	FGDG
	/%	/	/wt. %			
C1	75	1:4	17.2	25.8	43	14
C2	75	1:5				
C3	75	1:6				
C4	78	1:4				
C5	78	1:5				
C6	78	1:6				
C7	81	1:4				
C8	81	1:5				
C9	81	1:6				

## Data Availability

The original contributions presented in this study are included in the article. Further inquiries can be directed to the corresponding author.
